# Ultra-Early Treatment of Neurosurgical Emergencies with Endoscopic Endonasal Approach: Experience from Three Italian Referral Centers

**DOI:** 10.3390/jcm12175471

**Published:** 2023-08-23

**Authors:** Pier Paolo Mattogno, Matteo Zoli, Quintino Giorgio D’Alessandris, Daniele Bongetta, Valerio Maria Caccavella, Mario Rigante, Giuseppe Maria Della Pepa, Diego Mazzatenta, Liverana Lauretti, Alessandro Olivi, Giannantonio Spena, Cesare Zoia

**Affiliations:** 1 Department of Neurosurgery, Fondazione Policlinico Universitario Agostino Gemelli IRCCS, 00100 Rome, Italy; 2 Programma Neurochirurgia Ipofisi-Pituitary Unit, IRCCS Institute of Neurological Sciences of Bologna, 40121 Bologna, Italy; 3 Department of Biomedical and Neuromotor Sciences (DIBINEM), University of Bologna, 40121 Bologna, Italy; 4 Department of Neurosurgery, ASST Fatebenefratelli Sacco, 20100 Milan, Italy; 5 Department of Otolaryngology, Fondazione Policlinico Universitario Agostino Gemelli IRCCS, 00100 Rome, Italy; 6 Neurosurgery, Fondazione IRCCS Policlinico San Matteo, 27100 Pavia, Italy

**Keywords:** neurosurgical emergencies, endoscopic technique, transnasal endoscopy, pituitary apoplexy

## Abstract

Purpose: the aim of this multicenter study is to preliminarily assess the role of the Endoscopic Endonasal Approach (EEA) in ultra-early (i.e., within 12 h) management of selected neurosurgical emergencies in terms of clinical and radiological outcomes. Methods: 26 patients affected by sellar/parasellar pathologies with rapid progression of symptoms were managed with EEA within 12 h from diagnosis in three Italian tertiary referral Centers from January 2016 to December 2019. Both clinical and radiological data have been collected preoperatively as well as post-operatively in order to perform retrospective analysis. Results: The average time from admission to the operating room was 5.5 h (±2.3). The extent of resection was gross-total in 20 (76.9%), subtotal in 6 (23.1%) patients. One patient experienced re-bleeding after a subtotal removal of a hemorrhagic lesion. Patients with a longer time from admission (>4 h) to the operatory room (OR) experienced stable impairment of the visual acuity (*p* = 0.033) and visual field (*p* = 0.029) in the post-operative setting. Conclusions: The Endoscopic Endonasal Approach represents a safe, effective technique that can be efficiently used with good results in the management of selected neurosurgical emergencies in centers with adequate experience.

## 1. Introduction

Advancements in medical technology continue to reshape the landscape of neurosurgery, allowing for innovative approaches to the management of various pathological conditions. Among these approaches, the endoscopic endonasal approach (EEA) stands out as a minimally invasive technique that has gained prominence in addressing sellar and parasellar region pathologies. However, its application in emergency neurosurgical cases has been limited due to the typically slow-growing and benign nature of these conditions within the sellar/parasellar region. This stands in contrast to disciplines like otolaryngology, where the EEA has become the primary choice for addressing paranasal and orbital emergencies [1].

While the use of EEA in emergency neurosurgical cases has been infrequent, there is growing recognition of its potential significance in certain acute scenarios. Specifically, the EEA could emerge as a valuable tool in managing select cases that exhibit rapid deterioration of neurological symptoms. These cases may involve intralesional hemorrhages, uncommon fast-growing neoplasms, or even infectious diseases. The critical challenge lies in determining the feasibility and effectiveness of implementing the EEA within an ultra-early timeframe of fewer than 12 h from symptom onset.

To address this pressing question, a preliminary multicenter study has been conducted. This study aims to systematically assess the viability of employing the EEA in the ultra-early phases of acute neurological symptom exacerbation linked to sellar region pathologies. By investigating the applicability and outcomes of this approach, the study seeks to shed light on whether the EEA can become a valuable asset in emergency neurosurgery. As the field of neurosurgery continues to evolve, the potential integration of the EEA into emergency cases has the capacity to redefine established practices and offer new avenues for managing acute neurological cases. This article delves into the context, challenges, and prospects surrounding the feasibility of endoscopic transnasal operations in emergency neurosurgical cases, emphasizing the need for evidence-based insights to guide clinical decision-making in this evolving landscape. It is important to clarify that the study’s intention is not to directly compare the EEA with traditional surgical methods in various neurosurgical emergencies. Instead, the core objective is to underscore the feasibility and efficacy of utilizing the EEA as an alternative option for specific urgent interventions.

## 2. Materials and Methods

A retrospective data collection and analysis were performed dealing with patients treated for a neurosurgical emergency with EEA from January 2016 to December 2019 at three Italian referral Centers (Department of Neurosurgery, Fondazione Policlinico Universitario Agostino Gemelli IRCCS, Rome; Neurosurgical Department, Fondazione IRCCS Policlinico San Matteo, Pavia; Pituitary Unit, IRCCS Istituto delle Scienze Neurologiche di Bologna).

The patients eligible for this study had all developed one or more worsening symptoms, causing them to be admitted to the emergency departments of the respective centers.

The inclusion criteria were: 1—the presence of worsening clinical symptoms within 24 h before admission (milder symptoms might have been present even before, but the patient must have experienced a significant worsening within the previous 24 h), 2—the diagnosis of a pathology of the pituitary, parasellar or anterior skull base areas, 3—surgical treatment by means of an EEA, 4—the time from ER admission to OR < 12 h, 5—availability of preoperative and follow-up (12 months) clinical, endocrinological (PRL, FSH, LH, estradiol, total testosterone, free testosterone, TSH, fT4, GH, IGF1, ACTH, cortisol), and radiological data (head CT scan and/or brain MRI).

All patients underwent a diagnostic head CT scan without contrast in the emergency department, followed by a brain MRI when a deeper definition was necessary (location, size and anatomical relationship with the surrounding structures). A head angio-CT would have been performed to rule out vascular pathologies if the neurosurgeon or the neuroradiologist had any doubts. Indication for surgery has always been discussed collegially among the neurosurgical staff. Lesions specimens of patients were reviewed and assessed according to the WHO 2016 classification of brain tumors [2]. Both clinical and radiological conditions of the patients were analyzed at follow-up: neurosurgical, endocrinological, ophthalmological, and clinical evaluations were taken at 1, 3, 6 and 12 months after surgery and then annually. MRI was performed after 3 months, then at regular intervals of 6–12 months, depending on the clinical, radiological and histological features.

### 2.1. Surgical Procedure

The surgical team, consisting of Neurosurgery and Otorhinolaryngology specialists, performed a bilateral paraseptal sphenoidotomy using a rigid 4 mm 0° scope. In cases where anatomical variations like septal spurs blocked direct access to the sphenoid sinuses, a septoplasty or ethmoidectomy was carried out. Otherwise, gentle bilateral lateralization of the middle turbinate was sufficient to access the sphenoid. Before enlarging the sphenoidotomy inferiorly, the mucosa of the superior choanal edge was subperiosteally dissected to preserve the septal branches of the sphenopalatine arteries, which are usually located beneath the tail of the superior turbinate. The intersphenoidal septa, rostrum sphenoidalis, and the posterior third of the nasal septum were removed to gain broad control over the sella. Using a diamond burr drill, the floor of the sella was carefully removed until the periosteum of the sella became visible. A wide opening of the anterior face of the pituitary was achieved by removing bone from one cavernous sinus to the other. The dura was then incised in a “U” or “crux” pattern using a sharp instrument. Whenever possible, an extracapsular dissection was performed to completely remove the lesion while preserving the normal gland and the pituitary stalk. Lesions material under pressure was carefully removed through the dura incision using ring curettes. A 45° scope was employed to identify any residual lesion laterally, within the cavernous sinus, and superiorly. In cases of intraoperative cerebrospinal fluid (CSF) leak, a skull base plasty was performed through a multilayer technique with fat and fascia lata, the gasket seal technique, or fat with septal mucosal flap coverage.

### 2.2. Statistical Analysis

The association between continuous variables and outcome of interest was explored with the Mann–Whitney U-test. Comparison of categorical variables between groups was performed with the chi-square test employing the Fisher exact test when appropriate. Continuous variables are reported as mean (±SD), categorical variables as absolute and relative frequency. A *p*-value cutoff of 0.05 with Holm–Bonferroni correction was applied, thus shielding against type 1 error in the setting of multiple comparisons. All statistical analyses were performed using JASP (version 0.13.1; JASP Team, 2020).

## 3. Results

Twenty-six patients (22 male and four female) were admitted to the emergency room of three tertiary referral hospitals from January 2016 to December 2019 for the rapid worsening of neurological symptoms related to a pituitary region lesion. All non-comatose patients received a preoperative visual examination performed by the neurosurgeon, evaluating visual acuity and assessing the presence of visual symptoms through manual tests. A CT scan was required for all patients, with 8 of them further undergoing an MRI examination. Baseline characteristics of the patients and visual symptoms at the time of admission are detailed in Table 1; we report no other cranial nerve deficit in all of the alert patients evaluated.

Most of the patients with drowsiness exhibit slightly increased ventricle dimensions on admission CT, but none of them have clear acute hydrocephalus. The three comatose patients also have slightly increased ventricle dimensions at the admission CT, and they all have a voluminous hemorrhagic lesion.

A complete preoperative hormonal examination was performed in 20 patients: altered hormones were found in 13 cases.

The average time from admission in the emergency room to the operating room was 5.5 h (±2.3). Gross total resection of the pathology was achieved in 20 (76.9%) patients, being subtotal in the remaining 6 (23.1%) patients. No post-surgical complications were observed during hospitalization, except for a re-bleeding of a hemorrhagic lesion.

Histological evaluation yielded 18 (69.2%) pituitary apoplexies (PA) (13 non-functioning and five secreting prolactin adenomas). The remaining eight patients were diagnosed as being, respectively, affected by: post-radiation osteoradionecrosis of the clivus for infiltrating squamous carcinoma (impending meningitis with pneumocephalus) [3], meningioma of the diaphragm sellae (rapidly worsening visual acuity), suprasellar abscess, orbital hematoma (painful exophthalmos and visual impairment), craniopharyngioma, hemorrhagic Rathke cleft cyst, sellar lymphoma and (iatrogenic) sellar abscess.

At the last follow-up (mean: 36.8 ± 25.7 months), 24 (92.3%) patients were still alive and in good clinical condition. Dealing with the endocrinological outcome, all patients with hyperprolactinemia normalized the PRL value; nine (34.6%) patients were suffering from hypopituitarism. Neither tumor recurrence nor progression were detected.

Patients experiencing no improvement of the visual function in the post-operative setting had a statistically significantly longer time from admission to the operating room (6.3 ± 2.4 h vs. 4.5 ± 1.8 h, *p* = 0.035) (Table 2). Patients with a time from admission to surgery longer than 4 h had a higher risk of not recovering visual acuity (*p* = 0.033, OR: 5.71, 95% CI: 1.15–28.35) and visual field defects(*p* = 0.029, OR: 7.50, 95% CI: 1.23–45.81) post-operatively. Moreover, patients undergoing MRI in addition to CT scan underwent surgery significantly later (mean difference: 1.59 h, *p* = 0.047). Nevertheless, the post-operative improvement of visual acuity and field in this subgroup was comparable to the group of patients with only CT.

The presence of preoperative decreased visual field at the moment of admission was significantly associated with post-operative improvement of both visual acuity (*p* = 0.040) and visual field (*p* = 0.022). Likewise, the presence of a visual acuity impairment at the moment of admission was associated with improvement of visual acuity post-operatively (*p* = 0.014).

In summary, the EEA was feasible in all cases, and there was no need to resort to “traditional” surgery. No post-operative complications were observed, except for one case of re-bleeding in a hemorrhagic lesion and nine cases of hypopituitarism. At the last follow-up, there were no instances of tumor recurrence or progression. The lack of improvement in visual function is associated with a longer time between symptom onset and surgical intervention.

### 3.1. Illustrative Cases

#### 3.1.1. Case 1

**Figure 1:**
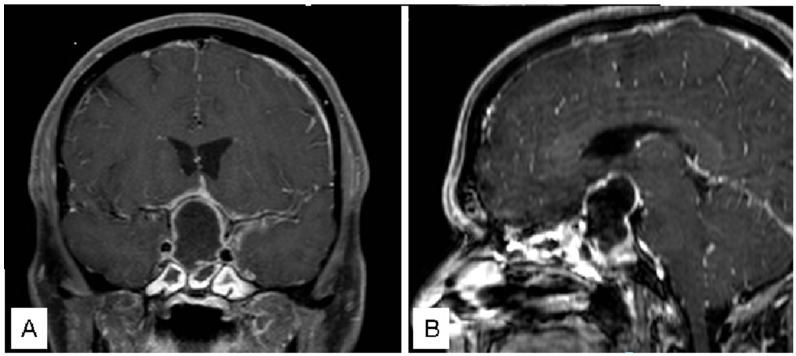
Coronal (**A**) and Sagittal (**B**) pre-operative T1 with gadolinium MR.

A 44 male patient with a sellar lesion was surgically treated with transsphenoidal surgery at another hospital. The lesion was identified as an “arachnoid cyst causing panhypopituitarism and visual deficit”. Seven days post-surgery, the patient presented with fever, headache, drowsiness, disorientation, and nuchal rigidity. Within 6 h of symptom onset, the patient was referred to our Emergency Department. Neuroradiological exams confirmed the presence of a sellar region abscess (Figure 1), necessitating emergency surgical evacuation that was achieved within 12 h of symptom onset.

**Figure 2:**
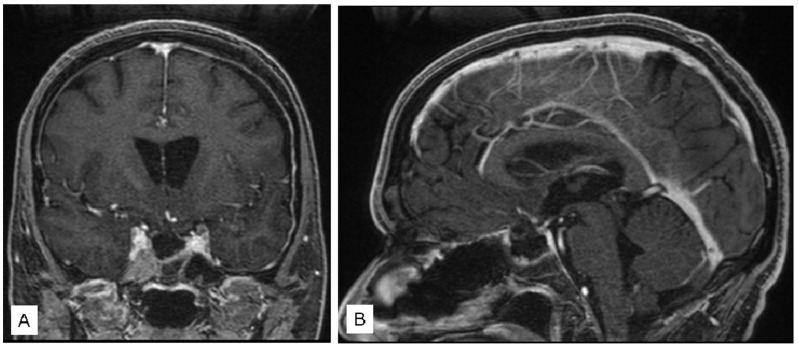
Coronal (**A**) and Sagittal (**B**) post-operative T1 with gadolinium MR.

Intraoperative cultures revealed the presence of Streptococcus constellatus. Subsequently, the patient received antibiotic therapy with Penicillin and Meropenem. Complete evacuation of the abscess was achieved (Figure 2), leading to an improvement in consciousness and headache.

#### 3.1.2. Case 2

**Figure 3:**
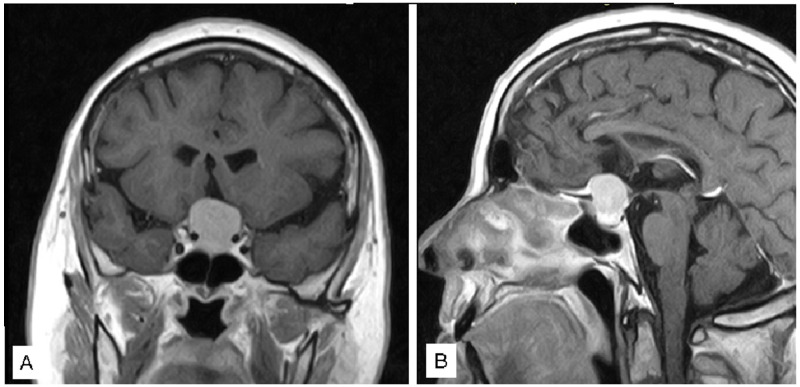
Coronal (**A**) and Sagittal (**B**) pre-operative T1 with gadolinium MR.

A 64-year-old female patient presented with severe bilateral visual impairment and bitemporal hemianopsia without hormonal disturbances. A head CT and a head MR (Figure 3) were performed; the patient underwent surgical intervention approximately 6 h after diagnosis.

**Figure 4:**
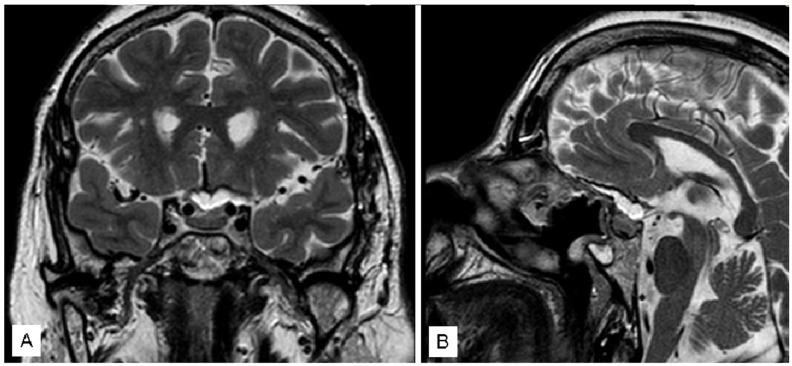
Coronal (**A**) and Sagittal (**B**) pre-operative T2 MR.

A near total resection, as showed by the post-operative MR (Figure 4), was performed, leading to a significant improvement in visual acuity immediately post-op.

Subsequent follow-up revealed a bilateral visual acuity of 10/10, with only right temporal hemianopsia remaining.

#### 3.1.3. Case 3

**Figure 5:**
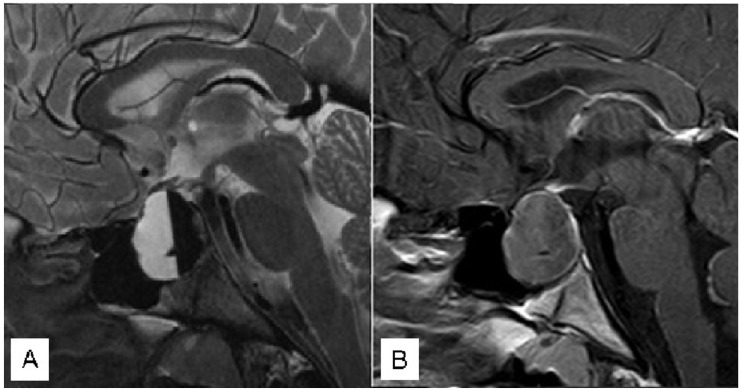
Sagittal pre-operative T2 (**A**) and T1 (**B**) with gadolinium MR.

An 18-year-old man went to our attention for acute onset of headache, blurred vision and asthenia. Immediately, he underwent a CT scan showing a sellar/suprasellar mass with a mixed signal: hypodense anteriorly and spontaneously mildly hyperdense posteriorly. Bio-humoral essays demonstrated hypopituitarism with mild hyperprolactinemia (60 ng/mL). Visual field performed in emergency showed bitemporal hemianopia. In the suspect of pituitary apoplexy, the patient underwent an MRI with gadolinium, confirming the pituitary hemorrhagic adenoma (Figure 5).

**Figure 6:**
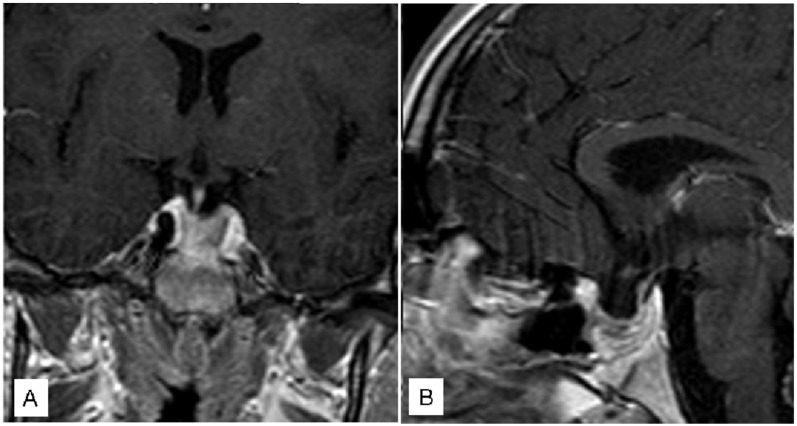
Coronal (**A**) and Sagittal (**B**) post-operative T1 with gadolinium MR.

He underwent an emergency endoscopic endonasal surgery with drainage of the hemorrhagic component of the tumor and removal of the mass. Histological examination confirmed the diagnosis of pituitary apoplexy. The post-operative course was uneventful, with immediate improvement of the visual deficit. The patient was discharged home 3 days later. At follow-up, the complete tumor resection was confirmed (Figure 6) with resolution of pre-operative visual symptoms.

After ten years, the patient is still under complete substitutive hormonal therapy for panhypopituitarism, and no recurrences have been observed.

## 4. Discussion

A definition of surgical emergency can be “a surgical procedure that cannot be delayed, for which there is no alternative therapy or surgeon, and for which a delay could result in death or permanent impairment of health” [4].

It is not always easy to distinguish between emergency, urgency and deferrable urgency. Nevertheless, in neurosurgical practice, it is universally recognized that the timing of surgery influences the outcome in rapidly worsening patients. While the timing cut-offs for pituitary emergencies may not be as precisely defined as those for stroke treatment [5], it is highly advisable to minimize the time between the onset of neurological deficits and surgical intervention [6]. Over the past years, EEA has earned widespread recognition among neurosurgeons as a leading approach for managing pituitary adenomas and skull base lesions, solidifying its status as a gold standard for selected pathologies [7,8,9,10]. Although this technique requires an adequate learning curve EEA offers several benefits, including enhanced visualization of delicate anatomical structures, reduced trauma to surrounding tissues, decreased post-operative complications, and shorter recovery periods. Its minimally invasive nature and precise maneuverability have contributed to improved patient outcomes and overall surgical success rates [11,12]. As a result, EEA has become a favored technique for addressing specific pituitary and skull base conditions, revolutionizing the field of neurosurgery.

Indeed, EEA has represented a revolution in the field of surgical techniques, and it has contributed to significantly expanding the surgical possibilities of treatment with the introduction of recent technological innovations (such as tailored and customized instruments, 3D and high-definition (HD) and 4 K monitors and magnification, neuronavigation systems) [13,14]. Noteworthy, EEA for urgent procedures is rather rare and is usually employed in case of an onset of rapidly worsening symptoms, such as acute neurological deficits, intense headaches, and deterioration in the state of consciousness. Those clinical manifestations are generally due to a pituitary or intralesional hemorrhage, more rarely to a rapid growth neoplasm or a severe infectious condition. Although the feasibility and safety of transcranial endoscopic procedures in emergency settings have been recently demonstrated [15], there are no large series yet in the literature about the management of urgencies in trans-sphenoidal endoscopic surgery [16,17], and almost all of them involve PA [18,19,20]. Our series confirms these data, reporting 18 patients (69.2%) affected by PA (13 non-functioning, five secreting prolactin). Moreover, there is still an open debate in the literature on the indications and management of PA. Surgery was traditionally considered the gold standard [21,22], but recently the role of conservative treatment has been re-evaluated, although some studies report largely conflicting results [23,24]. In a recent systematic review with meta-analysis comprising 14 studies including 457 cases (259 surgical treatments and 198 conservative treatments), Goshtasbi et al. stated that there are no significant differences in visual and endocrine outcomes in surgical versus conservative management of PA [19]. Still, the UK guidelines on the management of PA state that “patients with severe neuro-ophthalmic signs such as severely reduced visual acuity, severe and persistent or deteriorating visual field defects or deteriorating level of consciousness should be considered for surgical management; (III, B)” concluding however that “surgery should be performed preferably within the first 7 days of onset of symptoms. (III, B)” [25]. More specifically, in their meta-analysis, Tu et al. suggest that surgery should be advocated in pituitary apoplexy patients with severe visual field and acuity deficits, whereas conservative management may be preferable for patients with slightly decreased visual acuity and diminished pituitary function [22]. Zaidi et al. report on a case series of 42 PA patients with good and rapid recovery of visual and pituitary function after surgery [26]. In their series, all patients developed rapidly progressive worsening of symptoms, in particular visual symptoms (18/26—69.2%), oppressive headache (22/26—84.6%) and deterioration of the state of conscience (12/26—46.2%). As far as surgical timing in the case of PA is concerned, no agreement is yet to be reached in the literature: the results are very conflicting, with some authors suggesting a potential benefit of early surgery [6,18,27,28], whereas other papers do not prove statistically different outcomes among the subpopulations [29,30]. In our series, a cut-off time from admission to surgery of 12 h was established as an inclusion criterion with an average time of 5.5 h (±2.3). To the best of our knowledge, there are no studies yet in literature taking into consideration ultra-early surgery (within 12 h of diagnosis) when treating PAs. A recent comprehensive meta-analysis reports no statistically significant differences in visual outcome between PA patients treated <7 days vs. >7 days from the diagnosis (97.8% vs. 84.8%, *p* 0.07) [31]. Different results have been reported by Bills and colleagues, who advocated the role of early surgery for PA with statistically significant improvements seen among patients with visual deficits, whereas no differences were reported between the subgroup treated within 3 days and that >4 days [32]. In contrast, Seuk et al. reported a statistically significant improvement in visual acuity and field outcome among PA patients operated within 48 h from the onset of symptoms [33]. Similarly, Woo et al. showed a similar improvement in terms of visual deficits with early surgery (<3 days) compared to a delayed one [34]. As for radiological diagnosis, Rajasekaran and colleagues claim that MRI is the radiological investigation of choice, confirming the diagnosis of PA in over 90% of the patients [24]. Still, CT is certainly the most commonly used imaging modality in an acute clinical setting: it can highlight a sellar mass in >80% of the patients, but it can diagnose a PA in only 21–28% of cases [35,36]. In our series, the urgent brain CT scan made it possible to perform a diagnosis in most cases (18/26—69.2%): it was deemed necessary to carry out an urgent MRI only in some selected cases (8/26—30.8%). Moreover, performing MRI in our series resulted in a delayed surgery (mean difference: 1.59 h, *p* = 0.047) even though it was not linked to a worsening of the visual outcome. It is remarkable that patients with a clinical-radiological diagnosis of PA (18/26—69.2%) had a shorter time before surgery than patients with other diagnoses (mean difference 1.9 h, *p* = 0.038), probably because PA causes intense headaches and rapidly progressive clinical signs.

Noticeably, operative timing appears to be particularly crucial: we found that surgery performed within 4 h from the admission was always linked to an improvement of the visual function. As a matter of fact, in our series, patients operated on later than 4 h from the admission did not improve their pre-operative visual deficits. These data are noteworthy and have never been reported on other papers, to the best of our knowledge.

This study has some limitations. First: the number of patients is small, and their baseline characteristics are not homogeneous. This is relevant since the natural history of each pathology might be different. Still, the aim of this study was mainly to assess the technical feasibility of an emergency EEA. Second: we considered the admission to the emergency room as the initial time point from which to evaluate the time to surgery. This may lead to different sources of bias: firstly, the effective onset of neurological symptoms may precede several hours the time to admission. Secondly, both the hour of the day and the day of the week may have played a significant role in the timing of actual admission to the emergency room (the well-known night- or weekend- effect for ER admission). Thirdly, the fact that our Centers are referral ones might have accelerated the timing of admission when compared to other peripheral Centers. Lastly, this study is centered solely on the feasibility and effectiveness of the EEA in neurosurgical emergency cases. The intention of the study is not to draw a direct comparison between the EEA and traditional surgery within emergency contexts. Rather, the primary goal is to underscore the viability and success of employing the EEA for urgent neurosurgical interventions.

The study encompasses an examination of the EEA’s feasibility across a range of cases without directly juxtaposing it with traditional surgical methods. While the investigation does not delve into a comparative analysis between these approaches in emergency situations, its core objective remains to highlight the EEA’s potential and efficacy within the realm of acute neurosurgical care.

Hence, bearing in mind these limitations, we here further specify the preliminary value of our analysis. Moreover, the endocrinological outcome has not been thoroughly reported, as we believe that a topical comparison in an etherogeneous group of pathology like ours was pointless.

## 5. Conclusions

The EEA stands as a viable and efficient option for effectively addressing specific neurosurgical emergencies within experienced medical centers. While additional studies are required to corroborate our initial findings, our firsthand experience highlights a significant observation: surgeries performed within a 4-h timeframe are linked to notable enhancements in visual symptoms, notably the improvement of visual acuity. This underlines the potential advantages that the EEA holds in managing time-sensitive neurosurgical exigencies, reinforcing its position as a valuable tool in emergency neurosurgical care.

A dedicated endoscopy team should always be available within neurosurgical centers that handle emergencies in order to provide this option for emergency cases, particularly those involving sellar or parasellar pathologies. Adequate training of both the medical and nursing teams is necessary to fully utilize the possibilities that the transnasal endoscopic technique offers to neurosurgeons, especially in urgent and emergency contexts.

## Figures and Tables

**Table 1:** Baseline parameters and symptoms at presentation of the 26 patients admitted for a neurosurgical emergency. Categorical and continuous variables are, respectively, reported as number of patients (%) and mean (±SD).

Parameter	Overall (n = 26)
Age (years)	53.9 (±18.4)
Sex (Male)	22 (84.6%)
**Symptomatology**	
Headache	22 (84.6%)
Visual field deficits	18 (69.2%)
Visual acuity deficits	14 (53.8%)
Nausea/Vomit	14 (53.8%)
Drowsiness	12 (46.2%)
Rhinorrhoea	5 (19.2%)
Meningitis	3 (11.5%)
Coma	3 (11.5%)
Amaurosis	3 (11.5%)
Oculomotor Deficits	3 (11.5%)
Pneumocephalus	2 (7.7%)
Photophobia	2 (7.7%)

**Table 2:** Outcome of Patients with visual impairment: univariate analysis. VA, visual acuity; VF, visual field.

Parameter	Overall (n = 26)	Postop Stable VA (n = 15)	Postop Improving VA (n = 11)	*p* Value	Postop Stable VF (n = 14)	Postop Improving VF (n = 12)	*p* Value
Preop VA deficits	14 (53.8%)	5 (33.3%)	9 (81.8%)	0.014 *	6 (42.9%)	8 (66.7%)	0.225
Preop VF deficits	18 (69.2%)	8 (53.3%)	10 (90.9%)	0.040 *	7 (50.0%)	11 (91.7%)	0.022 *
Previous surgery	7 (26.9%)	4 (26.7%)	3 (27.3%)	>0.999	3 (21.4%)	4 (33.3%)	0.495
Time to op	5.5 (±2.3)	6.3 (±2.4)	4.5 (±1.8)	0.035 *	6.2 (±2.4)	4.8 (±1.9)	0.111

*: To underline the statistical significance.

## Data Availability

All data are available on request.
